# Muscle activity during simulated work in the cold

**DOI:** 10.1186/2046-7648-4-S1-A113

**Published:** 2015-09-14

**Authors:** Julie Renberg, Per Øyvind Stranna Tvetene, Øystein Nordrum Wiggen, Karin Roeleveld, Mireille Van Beekvelt, Hilde Færevik

**Affiliations:** 1 Department of Health Research, SINTEF Technology and Society, Trondheim, Norway; 2 Department of Neuroscience, Faculty of Medicine, Norwegian University of Science and Technology, Trondheim, Norway

## Introduction

The abundance of natural resources in the Barents region is encouraging growth and development in the far North, exposing more workers to outside work in the cold (average temperature and wind velocity at Rognsundet in Finnmark last winter were -2.6 °C and 9.8 m.s^-1^_, _with extreme values of -15.3 °C and 27.5 m.s^-1^). The aim of this study was to investigate the effect of realistic cold exposure on muscle activity, while wearing the cold-weather protective clothing used in the mining industry.

## Methods

15 male volunteers performed simulated work at two ambient temperatures (T_a_): -15 °C and 5 °C. The experimental protocol consisted of five test periods and four work periods with a total exposure time of two hours. The five 10-minute test periods started with a dynamic wrist flexion (DWF) exercise, followed by maximal voluntary contraction of the wrist flexor, elbow flexor and shoulder abductor. The four work periods consisted of manual work above head level (5 min), manual work at hip height (5 min) and a lifting exercise (5 min). EMG was used to measure muscle activity, and near-infrared spectroscopy (NIRS) measured local muscle metabolism. Heart rate (HR), skin and rectal temperatures (T_re_) were measured continuously. The protective clothing worn was identical under both environmental conditions.

## Results

**Figure 1:**
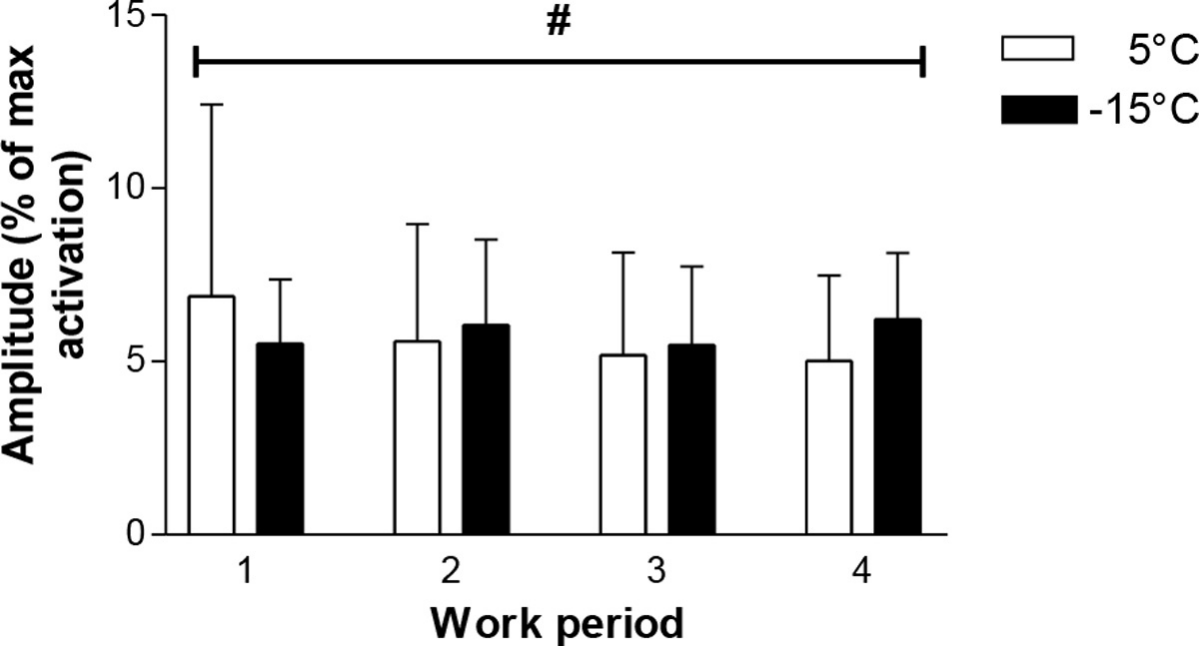
**Mean amplitude (% of max activation) in right flexor digitorum superficialis: manual work hip height for work period 1, 2, 3, and 4**. # significant interaction effect between T_a _and time. Data is presented as mean (SD) (N = 13).

During the two hours of exposure at -15 °C compared to 5 °C, mean skin temperature and finger skin temperature fell by 2.7 °C and 10-15 °C, respectively. Forearm skin temperature was stable at around 32.5 °C and 30.9 °C in 5 °C and -15 °C, respectively. No differences in either T_re _or HR between the T_a _were observed. There was a significant interaction effect between T_a _and time at the experiment in EMG activity (Figure 1). Deoxygenation during DWF was significantly more pronounced at 5 °C than -15 °C, but no interaction effect was found between T_a _and time.

## Discussion

Even though there was not a large difference in skin temperature at -15 °and 5 °C, it may have been sufficient to affect muscle function. At 5 °C, there was a gradual reduction in EMG activity, compared to a stable development at -15 °C. This difference could be related to temperature-dependent co-activation of muscle pairs [1]. More pronounced cooling would probably have had a more detrimental effect on muscle performance.

## Conclusion

This study has demonstrated that realistic cold exposure reduces skin temperatures, particularly in the extremities, while wearing protective clothing used in the mining industry. Exposure to -15 °C led to increased local muscle activation during manual work at hip height. While the current protective clothing provides sufficient thermal protection with regard to maintaining core temperature, the extremities are liable to become cold. It is therefore, important to focus on cold protection in the extremities in order to maintain miners' comfort and performance.
